# Development of an ovine efferent mammary lymphatic cannulation model with minimal tissue damage

**DOI:** 10.1186/s12917-016-0908-0

**Published:** 2016-12-12

**Authors:** Hung-Hsun Yen, Elizabeth Washington, Wayne Kimpton, Evan Hallein, Joanne Allen, Silk Yu Lin, Stuart Barber

**Affiliations:** Faculty of Veterinary and Agricultural Sciences, The University of Melbourne, Parkville, VIC 3010 Australia

**Keywords:** Lymphatic, Cannulation, Ewe, Model, Mammary, Ovine

## Abstract

**Background:**

Two mammary lymphatic cannulation models in sheep have been described with minimal use in the past 50 years. The purpose of this study was to investigate a new surgical technique to allow long term monitoring of mammary lymph flow and composition from the mammary glands, with rapid ewe recovery and minimal complications post-surgery.

**Results:**

We developed a modified methodology for cannulating the efferent mammary lymphatic from the mammary lymph node with minimum tissue damage. Compared to the previous models, our method required only a small incision on the aponeurosis of the external abdominal oblique muscles and thus reduced the difficulties in suturing the aponeurosis. It allowed for lymph collection and assessment for at least one week post-surgery with concurrent milk collection.

**Conclusion:**

This method allows for good ewe recovery post-surgery and in vivo sampling of efferent mammary lymph from the mammary lymph nodes in real-time and comparison with milk parameters.

## Background

An improved understanding of immunological responses in the mammalian udder during disease processes requires an ability to collect lymph from the infected gland over multiple time points, with the collection method causing minimal interference with the animal. One method to achieve this is via surgical cannulation of either afferent lymphatic ducts that lead to mammary lymph nodes, also called supra-mammary lymph nodes, or from efferent lymphatic ducts that drain lymph from these lymph nodes [1–3].

Surgical cannulation of the lymphatic vessels provides an approach for harvesting lymph draining the target tissues for biomedical research in many species including rats [4], humans [5], sheep [2], goats [6], cattle [7], mice [8] and dogs [9]. In sheep, a number of lymphatic catheterisation models for accessing pseudo-afferent or efferent lymph from different lymphatic vessels have been described, including the hepatic lymphatic [2], the efferent lymphatic of the mammary lymph nodes [2, 6], the prefemoral lymphatic [10], the popliteal lymphatic draining the lower hind limb [11], the efferent duct of the prescapular (superficial cervical) lymph nodes [12], intestinal lymphatic draining the small intestine [2, 13], the tracheal trunks draining oro-nasal regions [14, 15], the thoracic duct with thoracotomy [2] and the thoracic duct without thoracotomy [16]. These ovine cannulation models are useful tools in immunological research, with the ability to study in vivo, long-term pathogen and host interplay over time [17].

Lymphatic cannulation models such as the prefemoral model have been broadly applied to many studies, whereas mammary lymphatic cannulation has not, with only a few published reports of its use [1, 3, 18] since the publication by Lascelles and Morris (1961) more than 50 years ago [2] and none following the alternative method by Linzell (1960) [6]. One of the reasons for the limited application of the model of Lascelles and Morris (1961) is that the surgical procedures require incising a large portion of the aponeuroses of the external and internal abdominal oblique muscles and rejoining the aponeuroses, which requires advanced surgical skills. Indeed, the authors mentioned, “It is important to cut through the aponeurosis of the external and internal oblique abdominal muscles carefully as this tissue is difficult to suture. Unless the incision is closed correctly, the underlying peritoneum is exposed and the peritoneal contents may herniate.” [2].

The development of an improved mammary cannulation model will facilitate investigation of sheep diseases and the use of sheep as a model for investigation of diseases in other species, such as cattle and humans. One example of a potential use for disease investigation is mastitis, as this is a relatively common disease with a variety of aetiological agents [19]. It is particularly important in farmed ruminants as a source of reduced animal welfare, production and profitability [20, 21]. The development of RNA-seq [22, 23] and recent findings of the presence of exosomes in various body fluids [24, 25] has expanded the potential benefit of this type of model to enable long-term RNA profiling and exosome monitoring in lymph. Here, we report the development of a modified mammary lymphatic cannulation model of sheep.

## Methods

### Animals

The cadavers of three sheep were collected after they had been euthanised at completion of animal trials not associated with this study at the Faculty of Veterinary and Agricultural Sciences animal facility. Dissections were performed on the cadavers to approach the aponeurosis of the external abdominal oblique muscles and identify the external pudendal vessels medial to the aponeurosis. The use of cadavers to develop a new approach to the mammary lymphatic duct prior to surgery on live animals was undertaken to reduce the use of live animals. The use of live ewes for mammary cannulation in this experiment was covered under University of Melbourne Faculty of Veterinary and Agricultural Science Animal Ethics Committee (AEC) number 1312857.2 and for prefemoral cannulation under University of Melbourne Faculty of Veterinary Science AEC approval number 251683.

#### Mammary cannulation

Four lactating East Friesian cross ewes, aged 2.2–2.3 years were purchased from a commercial dairy. Three ewes were on their second lactation and one on her first lactation. The ewes had been lactating between 94 and 127 days prior to arrival at the animal house and averaged 1.05 litres milk produced per day post lambing. The ewes were transported to the animal house and fed on a mixed lucerne and oaten chaff ration (50:50) *ad libitum* for the first four days. They were then fed a mixture of this chaff and manufactured sheep pellets (Rumevite, Ridley Corporation, VIC, Australia) for the duration of the trial. They were milked by hand twice daily after arrival and acclimatised for a minimum of seven days before surgery. Prior to each milking the teat ends were disinfected with 70% ethanol soaked swabs. Milk volume was determined and 30 mL of milk was set aside for cell count and component analysis following the first few squirts of milk. An 18 mg bronopol tablet (Broad spectrum microtabs, Advanced Instruments) was added to the 30 mL tube to allow samples to be sent for analysis weekly with milk refrigerated within 1 h of collection. Measurements of milk cell count and components (fat, protein, solids-not-fat (SNF) & lactose) for each udder half were performed at Dairy Technical Services (North Melbourne, VIC, Australia) using a CombiFoss 5000 with standard FOSS reagents and technique, using International Dairy Federation standards.

#### Prefemoral cannulation

An additional three ewes of matched ages to those with mammary lymphatic cannulation had prefemoral efferent lymphatic ducts cannulated in order to compare the lymphocyte outputs and subsets between the two lymphatic circulations. These ewes were housed and fed under the same conditions as the ewes with mammary cannulations.

### General surgical procedures

On the day prior to surgery for mammary cannulation, ewes were fasted overnight and provided water *ad libitum* until the time of surgery. Anaesthesia was induced by intravenous injection of 1.0–1.5 mg thiopentone sodium (Boehringer Ingelheim, Australia) in 20–30 mL distilled water per sheep and then maintained with isoflurane (1.5–2.5%) and oxygen following intubation. The general surgical procedures and method for securing the bottles for lymph collection has been described in previous publications [14, 16]. We used clear vinyl cannulae (internal diameter 0.58 mm; external diameter 0.96 mm, Dural Plastics, Australia) coated with bioactive heparin (CBAS, Carmeda AB, Stockholm, Sweden) for all lymphatic cannulation surgeries. The bottles for lymph collections were secured on an animal with two tubular elastic net bandages (size 6, Surgifix, Australia). Each ewe was administered one injection of Temgesic (2.2 mg/kg) intramuscularly per day for the first two days post-surgery. Following cannulation, ewes were maintained on *ad libitum* feed and water in individual pens in sight of other ewes.

The presurgery and anaesthetic protocol for ewes undergoing prefemoral surgery was as per mammary cannulation surgery. The method for cannulating the prefemoral efferent lymphatic ducts of sheep was first described by Hall [10]. Briefly, an incision of about 10 cm was made through the skin and cutaneous muscle from the tuber coxae along the anterior border of the thigh. Subcutaneous fat was divided by blunt dissection then the anterior border of the tensor fascia was retracted to expose the circumflex iliac blood vessels and the associated prefemoral efferent duct. The detailed procedure used in cannulating this duct was identical to that used for the mammary efferent lymphatic duct as described later in this paper.

### Collection and analysis of mammary and prefemoral lymph

Lymph was collected twice daily in sterile 100 or 250 mL polypropylene collection bottles (Plastilab, Kartell Labware, Noviglio Italy) containing 1000 or 2000 IU of heparin (Pfizer). The bottle was fixed to netting surrounding the sheep’s abdomen to avoid inadvertent removal of the bottle or tube. The bottle was tethered to the inner tubular netting bandage using strings and held between the two tubular bandages. At each lymph collection the bottle was removed and replaced with a clean, sterile bottle containing heparin. To change the bottles, the cannula was removed from a small opening in the cap of the bottle before untying the bottle. The fresh bottle was secured to the netting with the same strings. The free end of the cannula was disinfected with 0.5% w/v Hibitane in 70% v/v alcohol before inserting it into the bottle through the small opening of the cap and sealed with adhesive tape. The total volume of lymph collected for each duct was measured and the average rate of lymph flow was determined.

Cells from a 50 μl sample of lymph were counted using a model Z1 Coulter Particle Counter (Beckman Coulter, USA). Cells in lymph were then washed 3 times in PBS containing 2% BSA, 0.4% EDTA and 0.1% azide (FACS wash) and stained for flow cytometric analysis of lymphocyte subsets. Monoclonal antibodies (mAb) against the T cell subsets CD4 (44–38), CD8 (38–65) and γδTCR (86D) were obtained from Dr Scheerlinck (Centre for Animal Biotechnology, The University of Melbourne) and have been described previously [26–28]. They were used as cell culture supernatants and detected with PE-conjugated sheep anti-mouse immunoglobulin (Ig) (Chemicon, Australia).

Cells were analysed fresh on a FACSCalibur Cytometer equipped with argon and red diode lasers (BD Immunocytometry Systems, USA). The instrument was calibrated with Calibrite Beads (BD Biosciences) and samples were collected and analysed using CellQuest Pro software (BD). Forward and side scatter were used to exclude dead cells.

## Results

### Baseline milk parameters of ewes before and after mammary cannulation

**Fig. 1:**
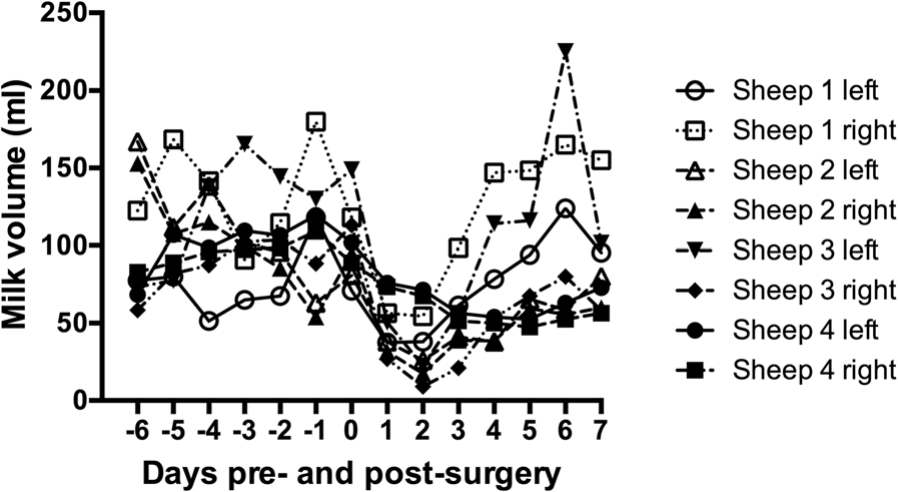
Milk production (half day) before and after mammary lymphatic cannulation surgery. The capacity for milk production in four ewes following mammary lymphatic cannulation surgery was monitored. Data represents the mean of milk production of each gland calculated from the twice-daily collections

For the first six days after arrival in the animal house prior to surgery, the average half-day milk production for a single gland from the ewes ranged from 51.5–180 millilitres. Milk was not collected from ewes in the evening following surgery. After surgery, the average half-day milk volume had an obvious drop in the first 3–4 days post-surgery in all sheep and then started to increase. At 4–7 days post-surgery, production levels of two ewes came back to similar quantities of that before surgery, but the milk production of the other two ewes remained at lower levels (Fig. 1). The quantities of milk collected were however adequate for milk quality tests.

We also monitored the amounts of fats, proteins, lactose and SNF in the milk before and after surgery. Daily fluctuations and individual differences on the percentages of these components in the milk were observed, but no obvious changes were noticed post-surgery. The corresponding range in percentages of fats, proteins, lactose and SNF in the milk were 6.54–10.02%, 5.86–8.4%, 4.36–5.1% and 12.03–13.38% before surgery and 7.04–10.13%, 5.92–8.57%, 4.53–5.17%, and 11.72–13.96% respectively post-surgery.

### Establishment of a modified mammary lymphatic cannulation

**Fig. 2:**
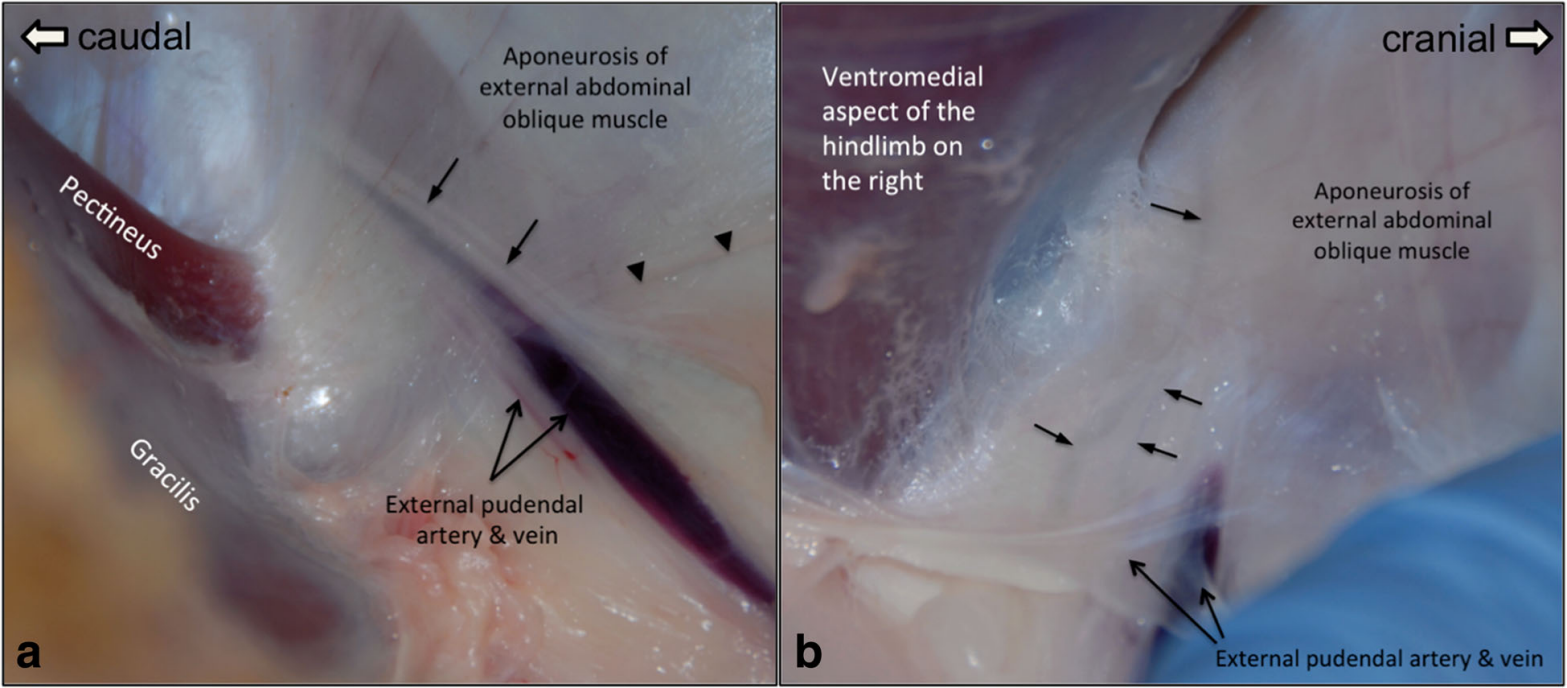
The dissection of ovine mammary lymphatics. The locations of the mammary lymphatics and their relationships to the external pudendal vessels found in different cadavers are depicted in (**a**) and (**b**). The mammary lymphatics leaving the mammary lymph centre usually ran together with the external pudendal vessels in parallel before entering the abdomen through the inguinal ring. **a** A mammary lymphatic medial to the aponeurosis of the external abdominal oblique muscle was located cranial to the external pudendal vessels in this sheep. The arrowheads mark the ventral edge of the aponeurosis of the external abdominal oblique muscle. →: mammary lymphatic. **b** Different to the findings in (**a**), the mammary lymphatic was caudal to external pudendal vessels in this animal with two lymphatic vessels identified. Figure 2**a** and **b** are positioned with cranial to the right and caudal to the left

**Fig. 3:**
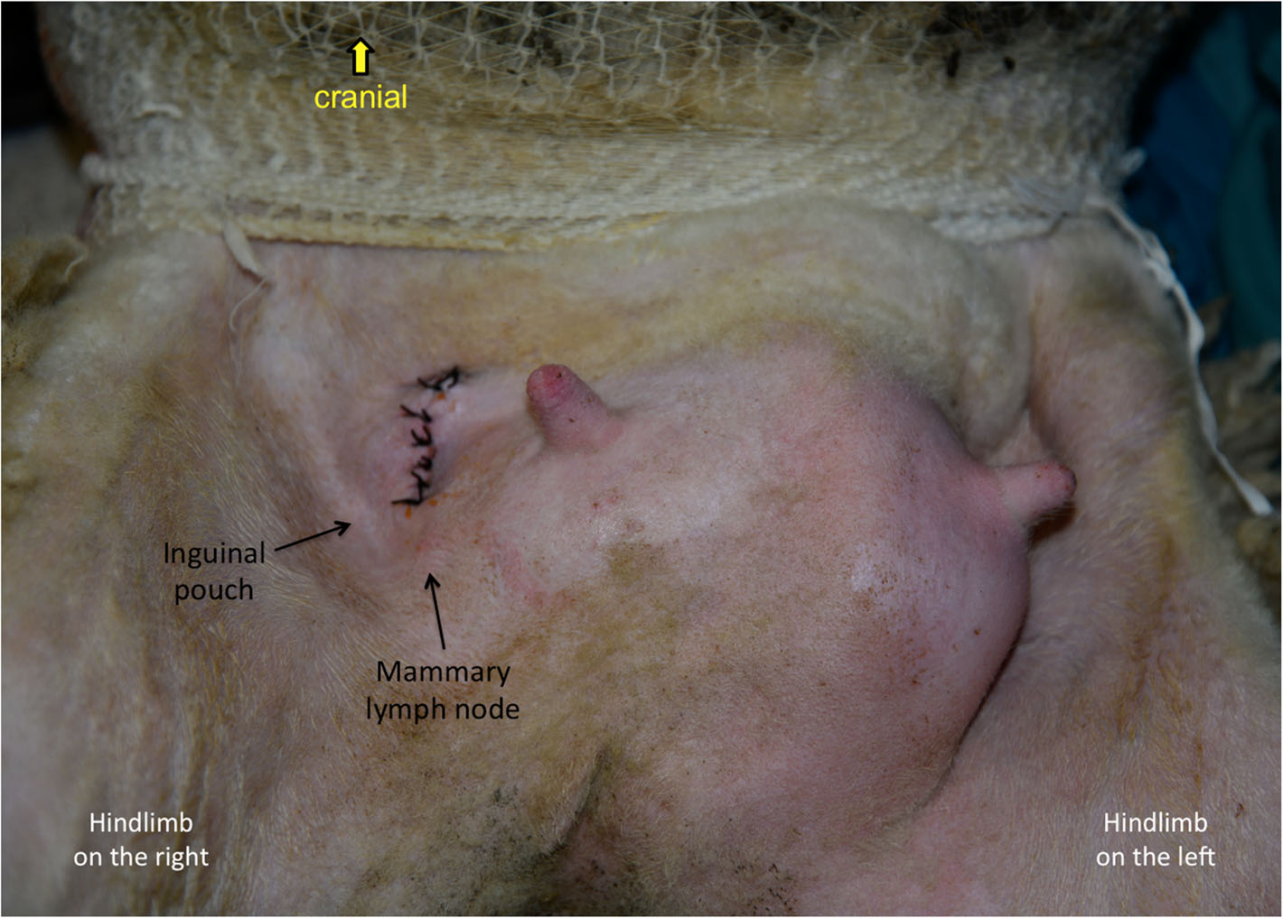
Site of the skin incision for accessing the efferent mammary lymphatic vessels. A skin incision of approximately six centimetres was made on the abdominal wall cranio-medial to the inguinal pouch to access the mammary lymphatic vessels. The skin incision was sutured post-surgery. The image is positioned with cranial to the top of the page

**Fig. 4:**
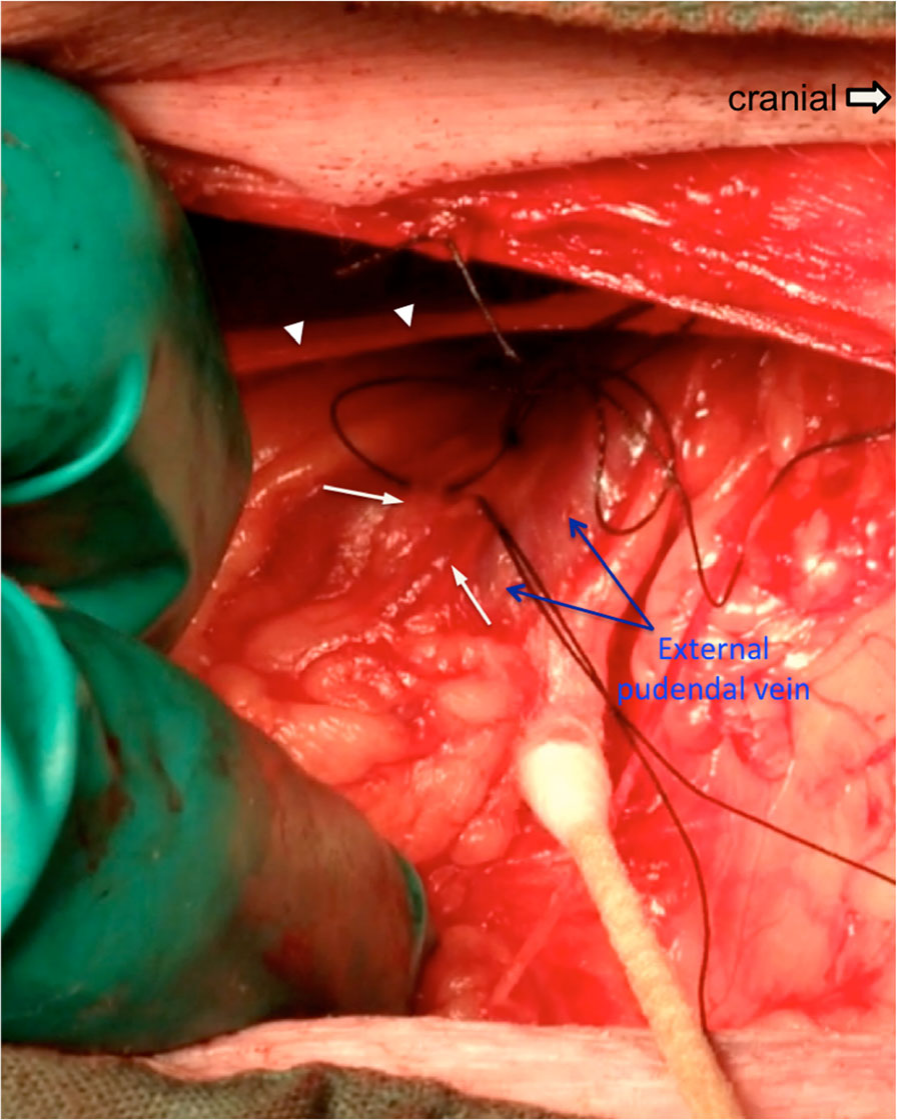
Intraoperative imaging of the mammary lymphatic vessels. The skin incision and the aponeurosis of the external abdominal oblique muscle were retracted using two fingers to expose the mammary lymphatic vessels coursing next to the external pudendal vein. Two mammary lymphatics of similar size caudal to the external pudendal vein on the right side of this animal were identified. The arrowheads mark the ventral edge of the aponeurosis of the external abdominal oblique muscle. The picture is positioned with cranial to the right

**Fig. 5:**
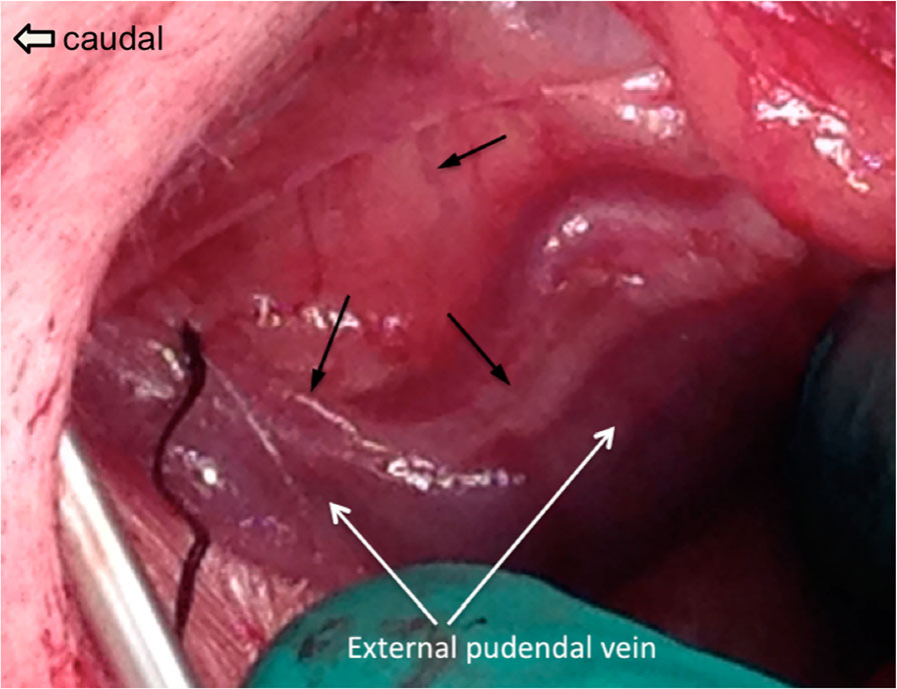
Intraoperative image showing adjoined mammary efferent lymphatic and external pudendal vein. A major mammary lymphatic ligated by silk suture was found to be adjacent to the external pudendal vein. The fibro-connective tissues strongly attached the lymphatic to the wall of the vein. A smaller mammary lymphatic running next to the vein was also found. The picture is positioned with cranial to the right. →: mammary lymphatics

Prior to live animal surgery, examination of cadaver anatomy showed that efferent mammary lymphatics coursed with the external pudendal vessels in parallel underneath the aponeurosis of the external abdominal oblique muscle entering the abdomen through the inguinal canal. The mammary lymphatics could have multiple branches and could be either cranial or caudal to the external pudendal vessels (Fig. 2). For live animal surgery a ventro-cranial to dorso-caudal orientated skin incision approximately 6 cm in length was made on the abdominal wall, cranio-medial to the inguinal pouch (Fig. 3). After skin incision, blunt dissection was performed to penetrate the subcutaneous fat and the superficial fasciae to approach the aponeuroses of the internal and external abdominal oblique muscles. After identifying the aponeuroses, a self-retained retractor was placed in the skin opening to generate an operating area. The lymphatics under the aponeurosis of the external abdominal oblique muscles were then identified as shown in a cadaver in Fig. 2. After identifying all branches of the mammary lymphatics, the external pudendal vessels and the lymphatics were detached from the aponeurosis of the external abdominal muscles using blunt dissection. By cutting through the caudoventral insertion of the aponeurosis of the external abdominal muscle to the fasciae connected to the rectus abdominal muscle, more space was created to access the segments of mammary lymphatics adjacent to the mammary lymph nodes. The surgical field was expanded by placing two fingers through the skin incision to increase the space to approach the lymphatic vessels caudal to the external pudendal vein (Fig. 4). Similar to the cadaver image in Fig. 2, multiple branches of similarly sized mammary lymphatic vessels were found during surgery (Fig. 4), while in other surgeries one mammary lymphatic vessel was tightly attached to the external pudendal vein (Fig. 5). This lymphatic vessel was the largest lymphatic branch in this surgery with a smaller lymphatic vessel next to the vein. We found the strong attachment of the largest mammary lymphatic branch to the external pudendal vein in two surgeries. It was necessary to identify all lymphatic branches and ligate them, with the largest lymphatic selected for cannula insertion.

**Fig. 6:**
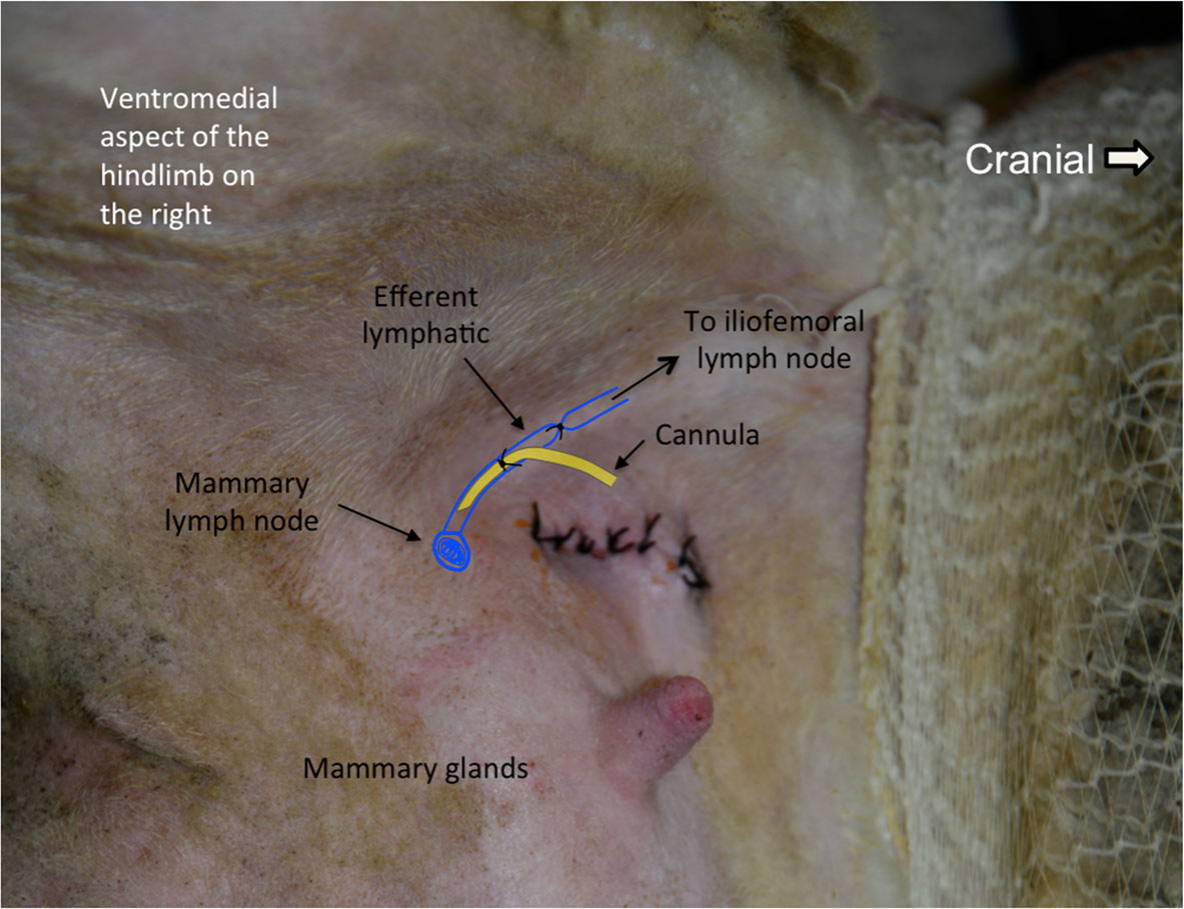
A schematic view of efferent mammary lymphatic cannulation. The sketch indicates the cannula placement location in the efferent mammary lymphatic vessel relative to the locations of the lymphatic ligation and a mammary lymph node adjacent to the right mammary gland. A segment of the efferent mammary lymphatic vessel near to the mammary lymph node was ligated using suture. The external pudendal vessels and lymphatics can show curved segments in the deep inguinal region, not demonstrated on this diagram. The efferent lymphatic vessels of the mammary lymph nodes (on both sides) enter the iliofemoral lymph node(s). The cannula was inserted into the lymphatic upstream from the ligation and secured with ligations (only one ligation is shown in this schematic picture). The sketch is superimposed over a photograph of a ewe post-surgery. The image is positioned with cranial to the right

The bevelled end of a cannula was placed beneath the aponeurosis of the external abdominal oblique muscle through a small stab incision at the dorsal part of the aponeurosis before inserting it into the lymphatic. The basic technique for inserting a cannula into a lymphatic has been described in previous publications [14–16]. Briefly, the procedures of cannula insertion into a lymphatic as shown in Fig. 6 were: 1. To place two pre sutures around the lymphatic upstream from its ligation, 2. To make a cut in the lymphatic using a pair of corneal scissors and then to insert the cannula into the lymphatic, 3. To secure the cannula in the lymphatic with the preplaced sutures.

After cannula insertion into the mammary lymphatic, the free end of the cannula was threaded through the skin near the wing of the ilium. The cannula was secured using a purse-string suture at its skin opening ventral to the wing of the ilium and craniodorsal to the pre-femoral lymph node(s) after exteriorising its free end. An additional suture was made to secure the cannula on the skin. Following surgery, the success of mammary lymphatic cannulation was confirmed in all ewes by the presence of blue dye in the cannula following an injection of 1 mL (0.5 g patent blue violet in 10 mL PBS) into the mammary tissues dorso-cranial to the teat and lymph dripping from the cannula. At the end of this study incisions on all ewes were healing well with no evidence of swelling and ewes were enrolled in a further study that required lymph collection.

### Prefemoral lymph cannulation

A single prefemoral efferent lymphatic duct was successfully cannulated in three additional non-lactating ewes.

### Cell profiles and lymph volume of mammary and prefemoral lymph

**Fig. 7:**
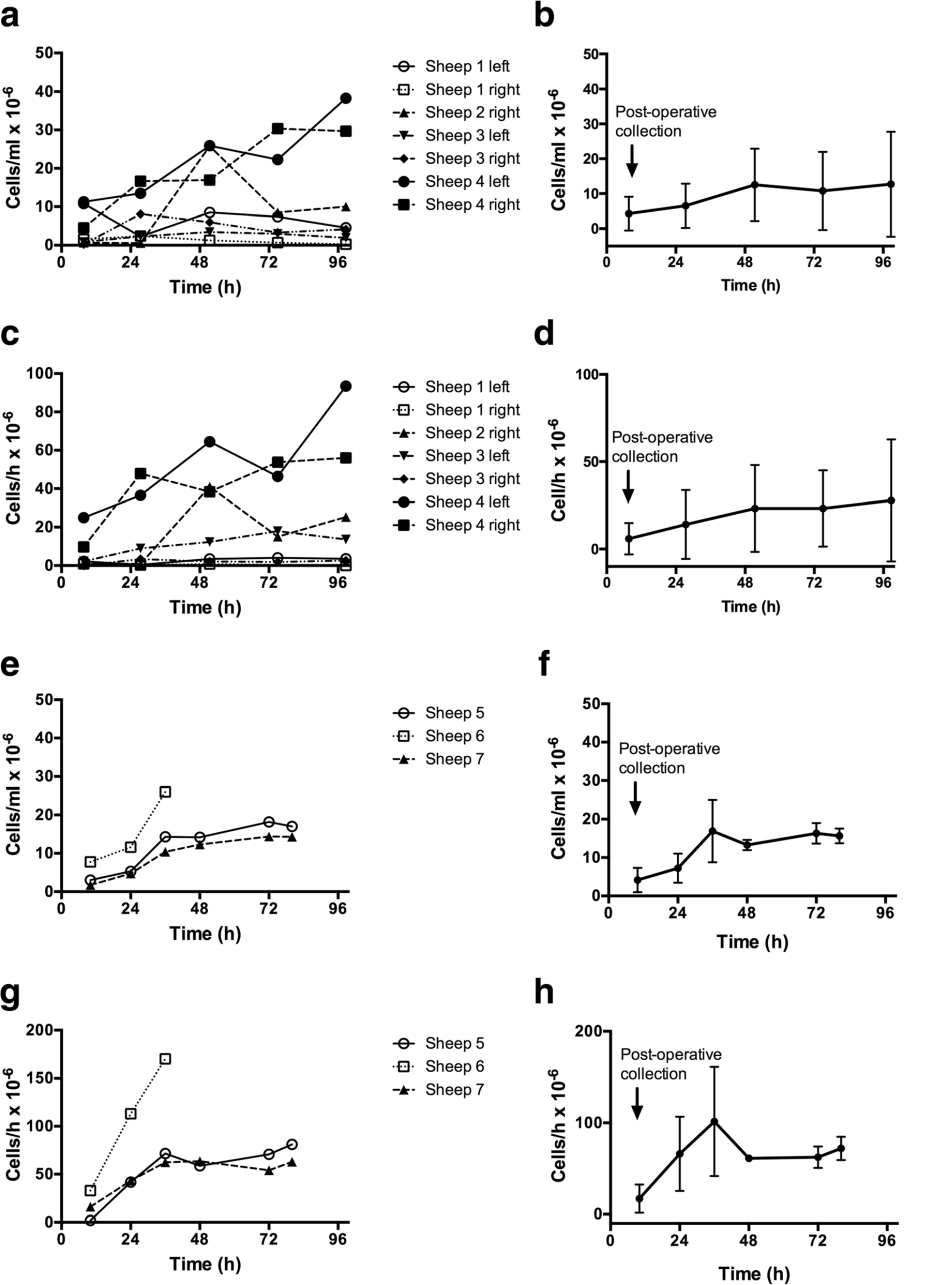
Lymphocyte concentration and output in efferent lymph from mammary and prefemoral lymph nodes. Comparison of cell concentrations and cell outputs in efferent lymph from mammary lymph nodes and prefemoral lymph nodes. **a**. Individual mammary cell concentrations. **b**. Mean mammary cell concentration ± standard deviation (SD). **c**. Individual mammary cell outputs. **d**. Mean mammary cell output ± SD. **e**. Individual prefemoral cell concentrations. **f**. Mean prefemoral cell concentration ± SD. **g**. Individual prefemoral cell outputs. **h**. Mean prefemoral cell output ± SD

We collected lymph from all eight cannulated mammary ducts and measured the rate of lymph flow, the cell concentrations and the cell output per hour. The presence of a low number of red blood cells was noted in the lymph samples for up to 4–5 days post-surgery. Lymph flowed well in four ducts with the flow rate ranging between 1.58 and 5.72 mL/h (daily volume, 37.8–205.7 mL) and the individual cell concentrations and outputs are shown in Fig. 7a and c respectively. Three ducts had slow, but continuous flow rates ranging from 0.09–1.07 mL/h. One cannulation completely blocked at day one post-surgery (Sheep 2 left side).

The flow of the three prefemoral efferent ducts ranged from 3–10 mL/h and the individual cells concentrations and outputs are shown in Fig. 7e and g respectively with the mean concentrations and outputs in Fig. 7f and h respectively. The drainage area of one prefemoral duct (Sheep 6) showed higher values than the other two cannulated sheep.

More than 97% of cells in the lymph samples were small lymphocytes, with the remainder large or blast-like lymphocytes. The phenotypes of small lymphocytes in mammary efferent lymph and those in the prefemoral efferent lymph of similar aged ewes are shown in Table 1. Over 80% of the lymphocytes in both the mammary and prefemoral efferent lymph were T cells (86% and 82% in mammary and prefemoral efferent lymph, respectively) with CD4+ T cells comprising 60% and 67% of the total T cells in mammary and prefemoral lymph respectively (Table 1).

**Table 1:** Phenotypes of lymphocytes in mammary and prefemoral efferent lymph

Subset	Mammary efferent lymph	Prefemoral efferent lymph
	Mean % ± SD	Mean % ± SD
CD4	51 ± 14	55 ± 7
CD8	15 ± 2	16 ± 6
γδ TCR	20 ± 9	11 ± 6

Cells were stained with monoclonal antibodies and analysed by flow cytometry. Data represents the phenotypes of lymphocytes from seven mammary efferent lymphatic ducts (lymph not available from one duct that blocked) and three prefemoral efferent lymphatic ducts. Cell subsets were compared between compartments using unpaired Mann–Whitney tests and no significant difference was found at *p* ≤ 0.05

## Discussion

The surgical procedures developed in this study provide a successful and less invasive approach for cannulating the efferent lymphatic vessels draining the mammary lymph nodes and the mammary glands. From the reports by Lascelles and Morris (1961) and the images in Fig. 2, it is clear that locating and accessing the mammary lymphatic vessel(s) directly from the dorsal portion of the aponeurosis of the external abdominal oblique muscle is easier since there are generally less adipose tissues in that region [2]. However, it is technically demanding and time-consuming to suture the aponeurosis and the sutured aponeurosis is likely to induce tangles of the cannula, in particular at the cannula’s insertion end in the lymphatic. The aponeurosis is a thin smooth sheet of fibro-connective tissue with specific orientations and arrangements of the tissue fibres. Damages of the aponeurosis can cause uneven healing of the connective tissues, resulting in an irregular shape of the aponeurosis. The strong pressure from the abdominal cavity on the damaged aponeurosis could alter its structure and shape even if it is properly sutured. Compared to the procedures by Lascelles and Morris (1961), to identify and access the segments of the efferent mammary lymphatics using our approach is more time-consuming since the efferent lymphatics course in the rich adipose tissues in the inguinal regions. However, to follow the segments of mammary efferent lymphatic vessels underneath the aponeurosis upstream, lymphatics in the inguinal regions can be located. To secure the success of the surgery and study, it is critical to confirm that all branches of the efferent mammary lymphatic vessels are identified and ligated. To find all lymphatic vessels in the adipose tissues in the inguinal region is the most challenging and time-consuming part of the surgery described in our study. However, our surgical procedures caused minimal damage to the aponeurosis of the external abdominal oblique muscle and the local tissues and are technically easier to perform compared to previous techniques. We suggest that the method described in this manuscript make the mammary lymphatic cannulation model more successful, combined with good animal recovery post-surgery.

Similar to that reported previously by Lascelles and Morris [1], we found variations in lymph flow volumes among different cannulated lymphatics. However, the total amount of lymph collected in our sheep each day (37.8–205.7 mL/day) are lower than that (450–900 mL/day) reported by Lascelles and Morris but similar to Watson and Davies [18]. Lascelles and Morris found that the stage of lactation, amount of milk production as well as the ewe’s activities correlated to lymph production. Our ewes were kept in metabolic cages with less exercise, were late in lactation and did not have lambs suckling them. This may explain why we harvested comparatively less lymph from the ewes in our study. A single cannula on one ewe blocked soon post-surgery in this study, however from our previous experience in other lymphatic cannulation surgeries this is a relatively common risk that lymph clots can completely block a cannula.

The cell concentrations in mammary and prefemoral efferent lymph were similar at around 10 × 10^6^/mL. The outputs of the prefemoral ducts (around 50–100 × 10^6^/mL) were higher than the mammary ducts (around 30–40 × 10^6^/mL), though there was wide variation in both. The cell output from a non-stimulated peripheral lymph node reflects the size of the node and hence blood flow, with the majority of lymphocytes derived from blood rather than afferent lymphatics [29, 30]. Both the prefemoral node, which weighs 2–3 g in an adult sheep, and the popliteal node, which weighs 1–2 g, have efferent outputs of around 50 × 10^6^/h [31]. The larger 3-6 g prescapular (superficial cervical) node has efferent outputs around 150 × 10^6^/mL [32]. It is difficult to relate the mammary node output directly to that of a peripheral node when the relative contribution of afferent lymph from the mammary gland is not also measured, especially as blood flow to the gland itself increases markedly during lactation [33].

The lymphocyte subsets in mammary efferent lymph were similar to prefemoral efferent lymph, indicating no difference between the mammary/mucosal and skin-draining efferent circulations in healthy sheep and typical of other non-stimulated adult efferent lymphatic ducts such as those draining the popliteal and prescapular lymph nodes [31, 32].

The ewes’ milk production following surgery is a key feature in the establishment of this lymphatic cannulation model. To maximise the applications of the mammary lymphatic models, it is necessary to confirm that samples from the lactiferous passages and alveoli and the lymph draining the inter-alveolar tissues can be harvested at the same time for comparative data analysis. The findings in our study verified that it is practical to collect and analyse the components in the milk following surgery. We suggest allowing the sheep to recover for at least 4–5 days post-surgery for milk levels and red blood cells in lymph to return to normal before receiving any further experimental treatments. Red blood cells may appear in efferent lymph in very small numbers due to the damage of capillaries that exist in the wall of the lymphatic vessels [11]. This may explain why we observed the presence of RBCs in lymph. In general, there should be no red blood cells in pure ovine efferent lymph. In addition to simply detecting the changes in milk samples in traditional mammary disease studies, the ability to monitor the responses in the lymph draining the gland can bring additional understanding of disease. In future work investigating the immune-biology of mammary disease and normal function, cannulation of the efferent duct could be combined with cannulation of the afferent mammary lymphatics to provide even more information from this model. Dendritic cells are present in ovine milk [34] and circulate to the mammary lymph node in afferent lymph, so by adding mammary afferent lymph [18, 35] to our model, we could obtain immune cells from three different compartments including antigen-presenting cells from the lactiferous passages (alveoli and canals), dendritic cells from the mammary inter-alveolar connective tissues and effector cells from the efferent mammary lymph of the same sheep. This further modification of the mammary lymphatic cannulation model would provide a powerful tool to examine the responses to disease.

## Conclusions

In conclusion, this improved cannulation technique enabled lymphocyte subset monitoring from ewes in late lactation for at least eight days following surgery and will be useful as a model to further study mammary disease and mucosal immunity. This model may also have significant application for monitoring vaccination or antibiotic performance at the level of the mammary gland.

## Abbreviations

SNF: solids-not-fat

## References

[1] Lascelles AK, Morris B (1961). The flow and composition of lymph from the mammary gland in merino sheep. Q J Exp Physiol Cogn Med Sci.

[2] Lascelles AK, Morris B (1961). Surgical techniques for the collection of lymph from unanaesthetized sheep. Q J Exp Physiol Cogn Med Sci.

[3] McKeever DJ, Reid HW (1987). The response of the supramammary lymph node of the sheep to secondary infection with orf virus. Vet Microbiol.

[4] Bollman JL, Cain JC, Grindlay JH (1948). Techniques for the collection of lymph from the liver, small intestine, or thoracic duct of the rat. J Lab Clin Med.

[5] Bierman HR, Byron RL, Kelly KH, Gilfillan RS, White LP, Freeman NE, Petrakis NL (1953). The characteristics of thoracic duct lymph in man. J Clin Investig.

[6] Linzell JL (1960). The flow and composition of mammary gland lymph. J Physiol.

[7] Hartmann PE, Lascelles AK (1966). The flow and lipoid composition of thoracic duct lymph in the grazing cow. J Physiol.

[8] Ionac M (2003). One technique, two approaches, and results: thoracic duct cannulation in small laboratory animals. Microsurgery.

[9] Uhley H, Leeds SE, Sampson JJ, Friedman M (1963). A technic for collection of right duct lymph flow in unanesthetized dogs. Proc Soc Exp Biol Med.

[10] Hall JG (1967). A method for collecting lymph from the prefemoral lymph node of unanaesthetised sheep. Q J Exp Physiol Cogn Med Sci.

[11] Hall JG, Morris B (1962). The output of cells in lymph from the popliteal node of sheep. Q J Exp Physiol Cogn Med Sci.

[12] Glover DJ, Hall JG (1976). A method for the collection of lymph from the prescapular lymph node of unanaethetized sheep. Lab Anim.

[13] Hein WR, Barber T, Cole SA, Morrison L, Pernthaner A (2004). Long-term collection and characterization of afferent lymph from the ovine small intestine. J Immunol Methods.

[14] Yen HH, Scheerlinck JP, Gekas S, Sutton P (2006). A sheep cannulation model for evaluation of nasal vaccine delivery. Methods.

[15] Schwartz-Cornil I, Epardaud M, Bonneau M (2006). Cervical duct cannulation in sheep for collection of afferent lymph dendritic cells from head tissues. Nat Protoc.

[16] Yen HH, Wee JL, Snibson KJ, Scheerlinck JP (2009). Thoracic duct cannulation without thoracotomy in sheep: A method for accessing efferent lymph from the lung. Vet Immunol Immunopathol.

[17] Hein WR, Griebel PJ (2003). A road less travelled: large animal models in immunological research. Nat Rev Immunol.

[18] Watson DL, Davies HI (1985). Immunophysiological activity of supramammary lymph nodes of the ewe during the very early phase of staphylococcal mastitis. Res Vet Sci.

[19] Contreras GA, Rodriguez JM (2011). Mastitis: comparative etiology and epidemiology. J Mammary Gland Biol Neoplasia.

[20] Hogeveen H, Huijps K, Lam TJ (2011). Economic aspects of mastitis: new developments. N Z Vet J.

[21] Bergonier D, de Cremoux R, Rupp R, Lagriffoul G, Berthelot X (2003). Mastitis of dairy small ruminants. Vet Res.

[22] Wang Z, Gerstein M, Snyder M (2009). RNA-Seq: a revolutionary tool for transcriptomics. Nat Rev Genet.

[23] Bonnefont CM, Toufeer M, Caubet C, Foulon E, Tasca C, Aurel MR, Bergonier D, Boullier S, Robert-Granie C, Foucras G (2011). Transcriptomic analysis of milk somatic cells in mastitis resistant and susceptible sheep upon challenge with Staphylococcus epidermidis and Staphylococcus aureus. BMC Genomics.

[24] Vlassov AV, Magdaleno S, Setterquist R, Conrad R (2012). Exosomes: current knowledge of their composition, biological functions, and diagnostic and therapeutic potentials. Biochim Biophys Acta.

[25] Robbins PD, Morelli AE (2014). Regulation of immune responses by extracellular vesicles. Nat Rev Immunol.

[26] Maddox JF, Mackay CR, Brandon MR (1985). Surface antigens, SBU-T4 and SBU-T8, of sheep T lymphocyte subsets defined by monoclonal antibodies. Immunology.

[27] Mackay CR, Beya MF, Matzinger P (1989). Gamma/delta T cells express a unique surface molecule appearing late during thymic development. Eur J Immunol.

[28] Maddox JF, Mackay CR, Brandon MR (1987). Ontogeny of ovine lymphocytes. II. An immunohistological study on the development of T lymphocytes in the sheep fetal spleen. Immunology.

[29] Hall JG, Morris B (1965). The origin of cells in the efferent lymph from a single lymph node. J Exp Med.

[30] Hay JB, Hobbs BB (1977). The flow of blood to lymph nodes and its relation to lymphocyte traffic and the immune response. J Exp Med.

[31] Kimpton WG, Washington EA, Cahill RNP (1990). Non-random migration of CD4^+^, CD8^+^ and gd^+^T19^+^ lymphocyte subsets following in vivo stimulation with antigen. Cell Immunol.

[32] Washington EA, Kimpton WG, Cahill RNP (1988). CD4^+^ lymphocytes are extracted from blood by peripheral lymph nodes at different rates from other T cell subsets and B cells. Eur J Immunol.

[33] Thompson GE (1980). The Distribution of Blood-Flow in the Udder of the Sheep and Changes Brought About by Cold-Exposure and Lactation. J Physiol London.

[34] Tatarczuch L, Bischof RJ, Philip CJ, Lee CS (2002). Phagocytic capacity of leucocytes in sheep mammary secretions following weaning. J Anat.

[35] Heath TJ, Kerlin RL (1986). Lymph drainage from the mammary gland in sheep. J Anat.

